# Neurosteroids in Schizophrenia: Pathogenic and Therapeutic Implications

**DOI:** 10.3389/fpsyt.2018.00073

**Published:** 2018-03-08

**Authors:** HuaLin Cai, Ting Cao, Xiang Zhou, Jeffrey K. Yao

**Affiliations:** ^1^Department of Pharmacy, The Second Xiangya Hospital of Central South University, Changsha, China; ^2^The Institute of Clinical Pharmacy, Central South University, Changsha, China; ^3^Medical Research Service, VA Pittsburgh Healthcare System, Pittsburgh, PA, United States; ^4^Department of Pharmaceutical Sciences, University of Pittsburgh School of Pharmacy, Pittsburgh, PA, United States; ^5^Department of Psychiatry, University of Pittsburgh School of Medicine, Pittsburgh, PA, United States

**Keywords:** neurosteroids, GABA_A_ receptor, metabolism, clinical trial as topic, schizophrenia

## Abstract

Neurosteroids are a group of important endogenous molecules affecting many neural functions in the brain. Increasing evidence suggests a possible role of these neurosteroids in the pathology and symptomatology of schizophrenia (SZ) and other mental disorders. The aim of this review is to summarize the current knowledge about the neural functions of neurosteroids in the brain, and to evaluate the role of the key neurosteroids as candidate modulators in the etiology and therapeutics of SZ. The present paper provides a brief introduction of neurosteroid metabolism and distribution, followed by a discussion of the mechanisms underlying neurosteroid actions in the brain. The content regarding the modulation of the GABA_A_ receptor is elaborated, given the considerable knowledge of its interactions with other neurotransmitter and neuroprotective systems, as well as its ameliorating effects on stress that may play a role in the SZ pathophysiology. In addition, several preclinical and clinical studies suggested a therapeutic benefit of neurosteroids in SZ patients, even though the presence of altered neurosteroid pathways in the circulating blood and/or brain remains debatable. Following treatment of antipsychotic drugs in SZ, therapeutic benefits have also been linked to the regulation of neurosteroid signaling. Specifically, the neurosteroids such as pregnenolone and dehydroepiandrosterone affect a broad spectrum of behavioral functions through their unique molecular characteristics and may represent innovative therapeutic targets for SZ. Future investigations in larger cohorts with long-term follow-ups will be required to ascertain the neuropsychopharmacological role of this yet unexploited class of neurosteroid agents.

## Introduction

The term “neurosteroid,” first introduced by Baulieu and Robel (1), refers to the steroids that are synthesized in the brain. The term “Neuroactive steroid” subsequently introduced in 1992 by Paul and Purdy (2) has a broader concept, which refers to those steroids that are produced by an endocrine gland and subsequently reach the brain through the bloodstream. Now the two terms coexist and are mutually complemented (3). Based on the differences in activity and structure, the neurosteroids can be categorized into the following classes: pregnane neurosteroids [allopregnanolone (ALLO) and allotetrahydrodeoxycorticosterone (THDOC)], androstane neurosteroids (androstanediol and etiocholanone), and sulfated neurosteroids [pregnenolone sulfate (PS) and dehydroepiandrosterone sulfate (DHEAS)] (4).

Since the neurosteroids are highly lipophilic, they can easily pass the blood-brain barrier and modulate neuronal excitability and functioning through interactions with ligand-gated ion channels and other cell surface receptors (4). Some of these steroids may exert additional effects on gene expression via nuclear steroid hormone receptors (4). Growing data lend further support to the notion that neurosteroid dysregulation plays a crucial role in the pathophysiology of several psychiatric disorders, and may be a therapeutic target for antipsychotic treatment (3, 5–8).

Moreover, some neurosteroids have been demonstrated to possess anxiolytic, antidepressant and/or antipsychotic properties in animals (9–11). At the cellular level, they may exert neuroprotective effects, such as stimulating neurogenesis or facilitating the regeneration of neurons after injury, as well as promoting myelination (12–14). In animal models, as evaluated by the performance on memory tasks (15–19), specific neurosteroids have also been linked to the cognitive function. Taken together, it is likely that neurosteroids may serve as a potential therapeutic target for attention deficit hyperactivity disorder, Parkinson’s disease, depression, anxiety disorders, and schizophrenia (SZ) (20–23).

Schizophrenia is a remarkably complex disorder with diverse behavioral symptoms and biological perturbations. Whether these alterations are independent biological processes or a combined result of a more fundamental pathology has yet to be determined. The dopamine hypothesis of SZ has been one of the most enduring ideas in psychiatry. It has been focused on the role of hyperdopaminergia initially, redefined to specify subcortical hyperdopaminergia with prefrontal hypodopaminergia subsequently, and characterized as a “final common pathway” recently, in the etiology of SZ (24). More recently, a more integrative view combines different neurotransmitter systems, in which glutamatergic, GABAergic (gama-aminobutyric acid), and dopaminergic pathways interact (25). Thus, hypofunction of the *N*-methyl-d-aspartate (NMDA) type glutamate receptor, possibly on critical GABAergic interneurons, may also contribute to the pathophysiology beyond dopaminergic dysfunction of SZ (26). In addition, many other theories have been proposed in SZ over the years that aim to characterize the inherent pathological processes related to altered neurotransmission and signal transduction, including autoimmune dysfunction, membrane phospholipids deficits, etc. (27–32). However, many metabolic pathways which are likely modified by current treatment and of what relevance they are to clinical outcome remain unclear.

Thus, the issue at hand, first, is to identify candidate biological process(es) that are associated with SZ. The complex roles of neurosteroids in bipolar disorder (33), major depression (22, 34), anxiety disorder (22), pain (35), epilepsy (36), and autism spectrum disorder (37) have already been extensively discussed in separate reviews. The present review explores a potential role of neurosteroids in a specific metabolic pathway (pregnenolone–progesterone–allopregnanolone pathway) that may provide innovative targets for therapeutic intervention and monitoring in SZ.

## Neurosteroids in the Brain

**Figure 1:**
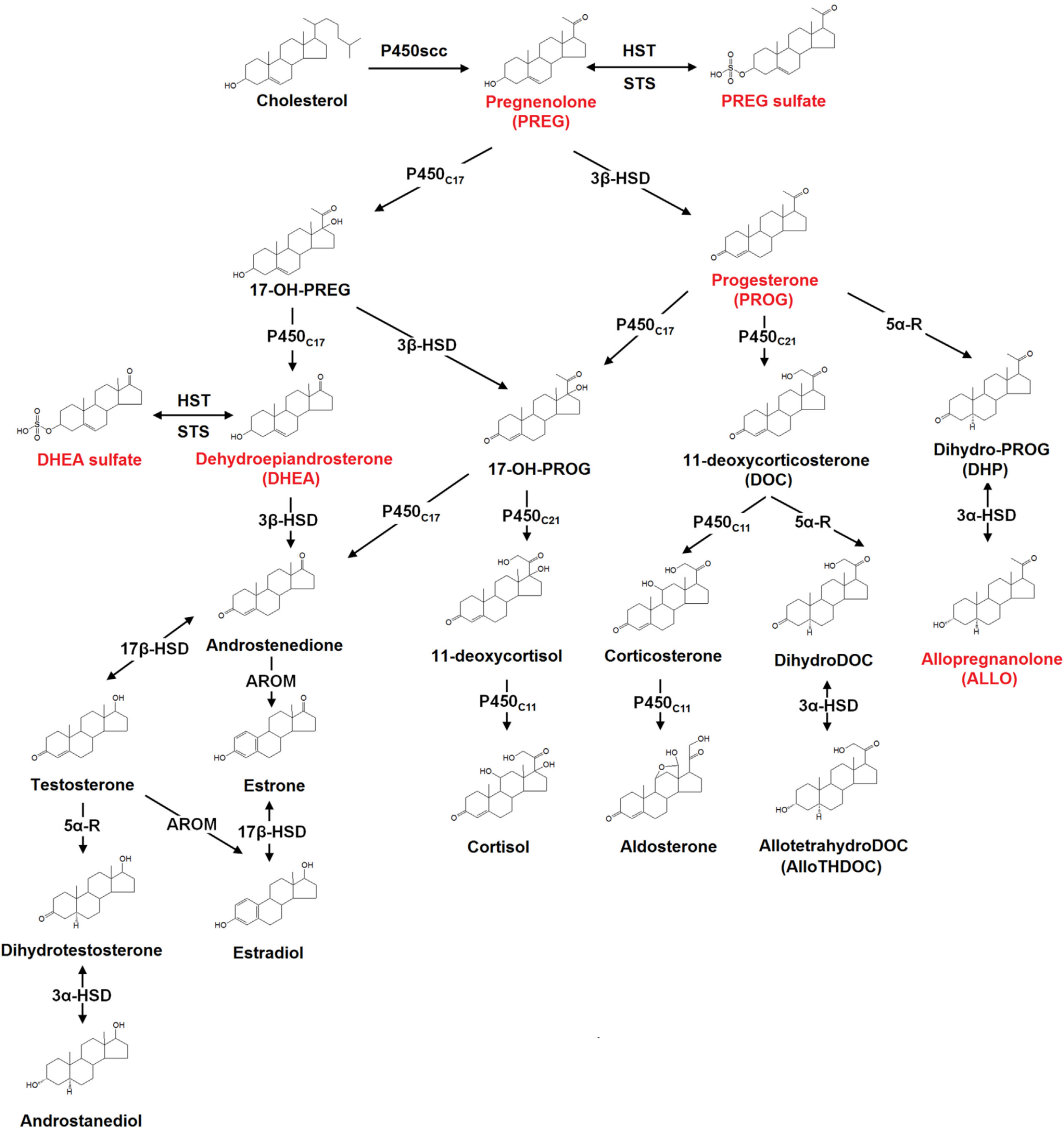
The biosynthesis pathways of neurosteroids in the human brain. Abbreviations: AROM, aromatase; HST, sulfotransferase; STS, sulfatase; P450scc, cytochrome P450 side-chain cleavage; P450c11, cytochrome P450 11β-hydroxylase/18-hydroxylase/18-methyl oxidase; P450c17, cytochrome P450 17α-hydroxylase/17,20-lyase; P450c21, cytochrome P450 21-hydroxylase; 3α-HSD, 3α-hydroxysteroid dehydrogenase; 3β-HSD, 3β-hydroxysteroid dehydrogenase; 5α-R, 5α-reductase; 17β-HSD, 17β-hydroxysteroid dehydrogenase. Those neurosteroids having the potential therapeutic roles in schizophrenia are highlighted in red in the figure.

Neurosteroids are biosynthesized in both the central and peripheral nervous systems, from cholesterol or through conversion of peripherally derived adrenal or gonadal steroids. The initial steps of *de novo* steroid biosynthesis include the translocation of cholesterol into the inner mitochondrial membrane and subsequent cleavage of cholesterol side-chain into pregnenolone (PREG) by enzyme CYP11A1 (cytochrome P450scc) (38). Conversion of cholesterol to PREG is the first rate-limiting step in the biosynthesis of all steroid hormones (38). The existence of several other key steroidogenic enzymes has now been identified in the human brain (38). These include the 3β-hydroxysteroid dehydrogenase, 17α-hydroxylase, 17,20-lyase, 17β-hydroxysteroid dehydrogenase (17β-HSD), 5α-reductase (5α-R), 3α-hydroxysteroid dehydrogenase, cytochrome P450 aromatase (P450arom), as well as hydroxysteroid sulfotransferase (HST) (38). Most of these enzymes are involved in *de novo* steroidogenesis in the brain. P450arom and 5α-R, however, are mainly responsible for the conversion in the brain of hormonal steroids originating from peripheral endocrine glands. The schematic diagram illustrating the neurosteroid pathways in the neuron or glial cell is shown in Figure 1.

Although locally synthesized neurosteroids play a major role as signaling molecules in the brain, the regulating mechanisms underlying the neurosteroid biosynthesis remain unclear. A previous study (39) indicated that the expression and activity of key steroidogenic enzymes in the brain may be regulated by adrenal and gonadal steroids, suggesting a putative association between peripheral and brain neurosteroids. Furthermore, their findings suggest that the neurosteroids in the periphery can serve as a proxy or surrogate marker for the regulation of neurosteroids in the brain. In fact, positive correlations between plasma and brain levels of neurosteroids have been demonstrated in rat (40, 41). A similar correlation has also been observed between plasma and CSF in humans (42).

## Mechanisms Underlying Neurosteroid Actions

Increasing evidence suggests that neurosteroids play a pivotal role in the development and functioning of the brain. In general, neurosteroids exert their genomic actions by targeting the intracellular receptors within the nucleus or cytoplasm. However, metabolites of progesterone (PROG) together with several other stress hormones can act on the membrane receptor through a non-genomic mechanism. In contrast to the genomic action of neurosteroids which is limited by protein synthesis, the modulation of the membrane receptor is fast occurring and only requires milliseconds to seconds (43). To date, it is well known that certain neurosteroids can non-genomically alter neuronal excitability in the brain *via* GABA neurotransmitter system (44). The receptors in several other neurotransmitter systems such as NMDA, dopaminergic, and sigma-1 receptors are also the targets for neurosteroids (45). The present review intends to underscore the critical role of neurosteroids [specifically, PROG and its 5α-reduced metabolites, as well as sulfated prognenolone and dehydroepiandrosterone (DHEA)] in the regulation of the neurotransmitter systems.

### Modulation of GABA_A_ Receptors

The GABA_A_ receptor is responsible for the primarily inhibitory currents in the brain (46). Neurosteroid actions at the GABA_A_ receptor complex have received increasing attention, given the extensive data published regarding the interactions between the specific neurosteroids and this receptor and its intimate involvement in different psychiatric illnesses.

**Figure 2:**
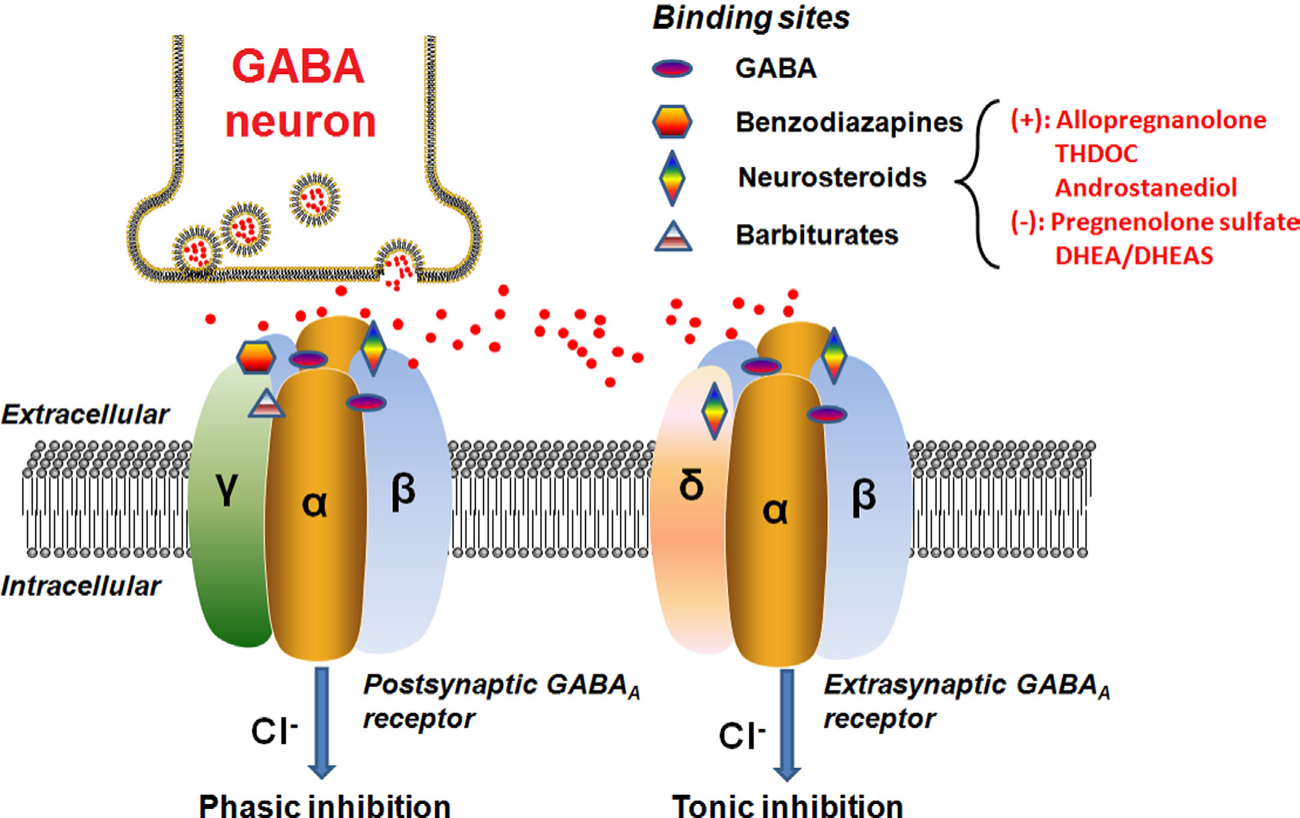
Schematic representation of the GABA_A_ receptor complex. Postsynatpic GABA_A_ receptors, which are pentameric chloride channels consisting of two α, two β, and one γ subunits, mediated the phasic GABAergic inhibition, whereas extrasynaptic GABA_A_ receptors, pentamers composed of two α, two β, and one δ subunits, primarily contribute to tonic inhibition. Neurosteroids activate both postsynaptic and extrasynaptic receptors to enhance the phasic and tonic inhibition, and thereby promote maximal net inhibition. The GABA_A_ receptors have distinct binding sites for numerous molecules, as illustrated in the figure. The typical neurosteroids that modulate positively (+) or negatively (−) on the GABA_A_ receptor complex at the synaptic membrane of nerve cells are indicated. The red dots represent the GABA neurotransmitter molecules released from the vesicles of the GABA neuron synapse. Abbreviations: GABA, γ-aminobutyric acid; THDOC, tetrahydrodeoxycorticosterone; DHEA, dehydroepiandrosterone; DHEAS, dehydroepiandrosterone sulfate.

Neurosteroids can rapidly alter neuronal excitability *via* direct interactions with GABA_A_ receptors and hence are often considered as endogenous modulators of GABA_A_ receptors in the brain (46–48). The GABA_A_ receptor is a ligand-gated chloride channel typically comprised of two α, two β, and either one γ or one δ subunits (Figure 2).

#### Binding Sites of GABA_A_ Receptor

The GABA binding site is located in the cleft between the α and β subunits (49). When activated by the endogenous neurotransmitter GABA, the neurons will be hyperpolarized through influx of chloride ions (49). Apart from the GABA site, there are many other binding sites clustered at the GABA_A_ receptor which are targeted by depressant/sedative agents, like benzodiazepines and barbiturates (49). Each pharmacological effect appears to be linked to a specific binding site on the receptor surface, depending on the subunit composition of the receptor. For instance, sedation has been linked to an enhancement at α_1_ subunit, whereas anxiolytic action is associated with the α_2_ subunit (50). Previous data have further shown that the α_5_ subunit largely contributes to sedative tolerance development to benzodiazepines and formation of associative memory and spatial learning (51–54), whereas the α_4_ subunit takes part in the regulation of anxiety (55) and the α_6_ subunit is relatively more responsive to pentobarbital (56) and neurosteroids (57). Alpha 1–5 subunits are also responsive to neurosteroids. In general, previous studies indicated that the neurosteroid (ALLO) enhanced GABA-evoked responses with all α subunit isoforms (α_1–6β1γ2_) (58, 59). As revealed by Belelli et al. (60), ALLO produced about sixfold to sevenfold enhancement of the GABA(EC_10_)-evoked response for α*_x_*_β1γ2_ (*x* = 1–5) type GABA_A_ receptors, whereas for receptors incorporating the α_6_ subunit, ALLO increased the current by 12-fold to the GABA(EC_10_)-evoked response. On the other hand, it should be noted that the neurosteroid sensitivity of α_4_ subunit containing receptor is also dependent upon the other partner subunits, as α_4_ receptors incorporating the δ subunit are highly steroid sensitive (60). By contrast, the isoform of the β subunit (1–3) has little impact on the GABA-modulatory actions of the neurosteroids (60).

The postsynaptic GABA_A_ receptors usually contain the γ subunit and are sensitive to both benzodiazepines and neurosteroids (44). Physically, they are ubiquitously distributed in the brain to generate the phasic currents in response to the GABA released from vesicle. The presence of the γ subunit within the GABA_A_ receptor is not a prerequisite for neurosteroid activities (60). Actually, a comparison of ALLO modulation of GABA responses mediated by α_1β1_ and α_1β1γ2_ receptors indicates that the former is more responsive, increasing the GABA-evoked response above the apparent maximal response to GABA (59, 60). The isoform of the γ subunit has little, or no effect on the maximal GABA-modulatory effect of ALLO, but significantly influences the potency of the neurosteroid, suggesting that the γ_1_ subunit containing GABA_A_ receptors are less effective than γ_2_ subunit containing GABA_A_ receptors (59, 60).

The extrasynaptic GABA_A_ receptors, however, are preferentially activated when GABA levels are low and ambient because of their high GABA affinity in comparison with the postsynaptic GABA_A_ receptors (61, 62). The extrasynaptic GABA_A_ receptors containing the δ subunit are expressed in various brain regions including the hippocampus, thalamus, amygdala, hypothalamus, and cerebellum (63). These extrasynaptic receptors generate non-desensitizing tonic inhibition currents that are highly sensitive to the extracellular GABA concentration (64). Tonic current controls the baseline excitability and generates shunting inhibition in the dentate gyrus, which is a critical region controlling afferent neuronal inputs and contributing to the formation of memories (46, 65, 66). Additional findings further indicate that the extrasynaptic receptors within specific brain regions (e.g., hypothalamus, hippocampal dentate gyrus, and cerebellum) are highly sensitive to neurosteroids, which may provide us with an important therapeutic target for neurosteroids (64). Initially, it was reported that the incorporation of the δ subunit reduced the GABA-modulatory actions of the neurosteroids (67). Subsequently, several studies demonstrated that the δ subunit greatly enhanced the steroid sensitivity of the receptor for some subunit combinations (60, 68, 69). The underlying reasons for these discrepancies are unknown. However, the δ subunit is known to associate with the α_6_ subunit in cerebellar granule cells and with the α_4_ subunit in thalamus and dentate gyrus granule cells of the hippocampus (60). Therefore, such tonic currents greatly influence neuronal excitability and hence may be an important locus of neurosteroid actions (70).

The exact neurosteroid binding sites on the GABA_A_ receptor remain to be characterized. It is not known whether γ and δ subunits have functional neuroteroid binding sites. However, the initial findings suggest at least two separate binding sites for neurosteroids consisting of an allosteric site within the α subunit and a direct activation site at the α-β subunit interface (71). Such distinctive binding sites further lead to the agonistic and antagonistic actions. For example, ALLO, allotetrahydrodeoxycorticosterone, and androstanediol are potent positive allosteric modulators of GABA_A_ receptors (4), whereas sulfated neurosteroids like PS and DHEAS have inhibitory actions on GABA_A_ receptors (4, 72). Further, the agonistic actions can be differentiated into direct activation or allosteric enhancement of GABA_A_ receptors (70, 73).

#### Modulation of GABA_A_ Receptor by Neurosteroids

The modulatory effects of neurosteroids on the GABA_A_ receptor are complex, and depend on the type and structure of the steroids, as well as on the location and subunit compositions of the receptors.

Neurosteroids have a unique action mode: at high concentrations (≥1 µM), neurosteroids directly activate GABA_A_ receptors to attain ceiling value by occupying all available neurosteroid binding sites, whereas at low concentrations (<1 µM), they allostericallly potentiate GABA_A_ receptor currents at a limiting value by fully occupying the allosteric site (44). Therefore, the overall net GABAergic inhibition contributed by neurosteroid-induced activation of GABA_A_ receptors may occur through allosteric and direct activation mechanisms. Despite increasing evidence shows that many neurosteroids and other medications can modulate GABA_A_ receptor functions, the relationship between the chemical structure of neurosteroids and their biological activities on the GABA_A_ receptor remains to be elucidated.

**Figure 3:**
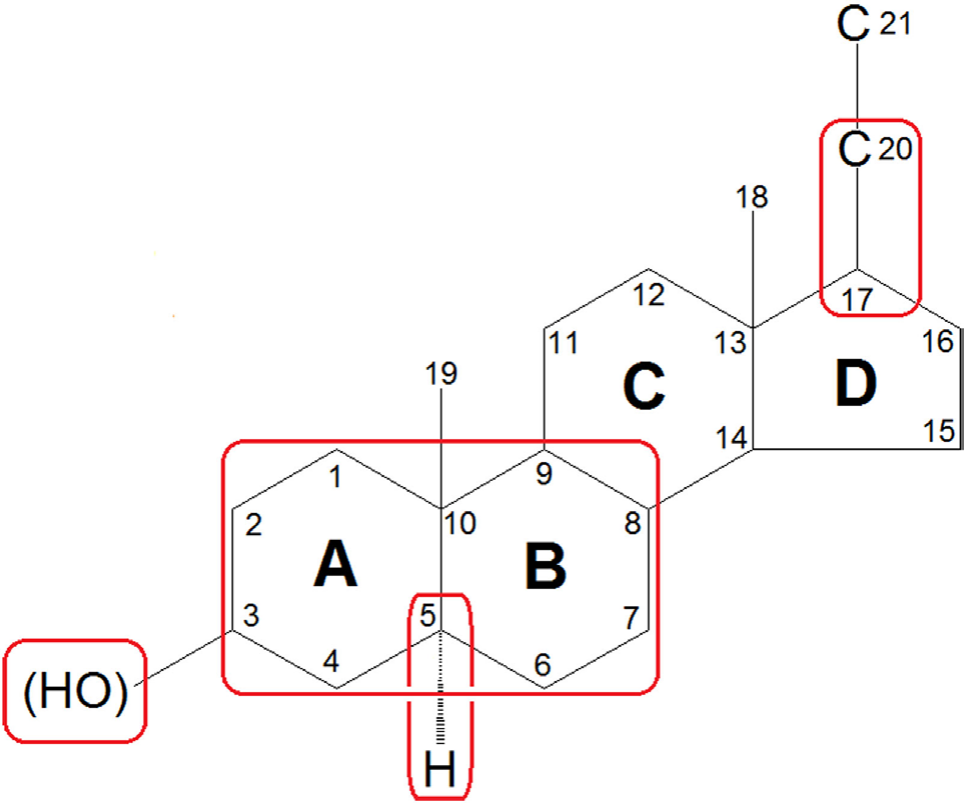
Features of general neurosteroid structure with the rings and carbon positions labeled. Hydroxyl groups are denoted β if they are oriented above the plane (solid line) and α if they are oriented below the plane (dashed line). GABA_A_ receptor-activated neurosteroids have the following structural requirements as highlighted with red rounded rectangle: (1) the scaffold geometry at the A/B ring; (2) a hydrogen-bond donator in C3 position which is indispensable for the binding affinity and the enhancing functions of neurosteroids; (3) the stereoselectivity of C5α-H which may affect the potency of the neuorsteroids; and (4) a hydrogen-bond acceptor in C20 position or a flexible bond at C17 position is important for high-potency positive allosteric modulation.

To date, systematic investigations of neurosteroids have identified the following structural characteristics that confer their activity at the GABA_A_ receptor (74–76): (1) the scaffold geometry at the A/B ring; (2) a hydrogen-bond donator in C3 position which is indispensable for the binding affinity and the enhancing functions of neurosteroids; (3) the stereoselectivity of C5α-H which may affect the potency of the neuorsteroids; and (4) a hydrogen-bond acceptor in C20 position or a flexible bond at C17 position is important for high-potency positive allosteric modulation (Figure 3). Currently, over 500 synthetic analogs based on neurosteroid structures have been screened for brain activity (77).

### Modulation of Glutamate Receptors

Previous studies have shown that the NMDA receptor can be regulated by specific neurosteroids (78, 79). The NMDA receptor possesses at least two distinct sites for neurosteroids, which differentially mediate the effects of positive modulators and the effects of negative modulators (80). Sulfated neurosteroids, such as PS and DHEAS, appear to be potent allosteric agonists on the NMDA receptor complex.

Initial data from electrophysiological experiments have also indicated that PS modulation of the NMDA receptor may be attributed to its ability to increase the frequency and duration of NMDA-activated channels opening (81). Subsequently, recombinant NMDA receptors with specific subunit compositions have demonstrated that mechanisms underlying the actions of PS on NMDA receptors are more complex. PS is able to potentiate NMDA receptor subunits GluN1/GluN2A and GluN1/GluN2B, as well as inhibit GluN1/GluN2C and GluN1/GluN2D subunits (82). Thus, the overall effect of PS on NMDA receptors will depend upon the binding affinities as well as the composition of NMDA receptor subunits (79). Since most available studies were carried out in neurons where NMDA receptor with GluN1/GluN2A and GluN1/GluN2B subunits are predominant, PS and DHEAS were often considered to be solely potentiating, rather than inhibiting compounds (79).

Additionally, PS is a typical representative member of neurosteroids that have indirect potentiating effects on NMDA receptors mediated through the inhibition of AMPA, kainate, and GABA_A_ receptors (83, 84).

### Modulation of Dopaminergic Activity and Sigma Receptors

Accumulating data suggest a role of the neurosteroids, especially PROG, in modulating the dopamine system either directly, or indirectly through the GABAergic system. Several previous studies have reported that: (1) a single dose of PROG can increase the levels of dopamine and its metabolites in the striatum of male rats (85) and the extracellular levels of striatal dopamine (86); (2) systemic administration of PROG also stimulates the release of dopamine in the striatum of intact male as well as ovarectomized female rats (86) and increases ethanol-induced mesocortical dopaminergic activity (87); (3) in women, PROG can increase the dopaminergic activity in amygdala (88); and (4) PROG also exhibited neuroprotective effects on the dopaminergic system of mice during degeneration (89).

On the other hand, other endogenous neurosteroids may modulate the dopaminergic signaling in opposite ways. Allopregnanolone diminishes dopaminergic neurotransmission through the modulation of basal and stress-induced dopamine release in the rat cerebral cortex and nucleus accumbens (90). Several other studies have demonstrated that increased brain levels of ALLO can dampen the release of dopamine in rat brain dopaminergic regions (90, 91). Similarly, benzodiazepine receptor agonists can suppress dopaminergic activity *via* GABA_A_ receptors in the striatum (92). Moreover, low doses of ALLO increase dopamine release, while higher doses decrease it, thereby exerting a dual modulatory effect on the dopaminergic nigro-striatal system (93). It is possible that via activation of the GABA_A_ receptor, ALLO directly influences dopaminergic transmission through intracellular signaling mechanisms such as activation of protein kinases (94, 95). Electrophysiological findings from rat brain slices lend further support that a bidirectional mechanism consisting of both GABAergic and glutamatergic inputs underlies the firing pattern of dopaminergic neurons (96).

Studies on binding affinity and pharmacological effects have revealed that certain neurosteroids (PS, DHEAS, and PROG) interact with sigma receptors, which are also present in high densities in the brain (97, 98). DHEAS and PS act as agonists, whereas PROG functions as an antagonist (99) on sigma receptors. Actions of PS and DHEAS on selective sigma-1 receptors may exhibit a potent modulating effect on excitatory neurotransmitter systems, such as the glutamatergic and cholinergic systems (98). Selective sigma-1 receptor ligands can modulate NMDA-mediated glutamatergic neurotransmission. This modulation plays a pivotal role in various neuroadaptational phenomena, including seizures, long-term potentiation, learning and memory, acute neuronal death, and neurodegeneration (99).

### Neurosteroid-Induced Neuroprotection

Although SZ is not conceptualized as a typical degenerative disorder, a large body of evidence suggests that, in a subset of SZ patients, glutamate-mediated excitotoxicity occurs in certain hippocampal and cortical areas (100, 101). Patients with SZ who have excitotoxic damage would be expected to present poor outcomes characterized by anatomical evidence of progressive neurodegeneration, pronounced negative symptoms and cognitive deficits, and profound psychosocial deterioration (102).

Neurosteroids have demonstrated neuroprotective effects in both central and peripheral nervous system by attenuating excitotoxicity, brain edema, inflammatory processes, oxidative stress, and neural degeneration (103). Additionally, neurosteroids accelerate and improve neurogenesis and myelination (103, 104). Previous findings have also advanced that PREG, a neurosteroid precursor, exhibits neuroprotective effects against glutamate-induced neurotoxicity (105), stabilizes microtubules (106), enhances polymerization and activates neurite outgrowth (107), and improves myelination (108). Meanwhile, the neuroprotective effects of both DHEA and DHEAS may be attributed to their modulatory effects on GABA_A_ receptors (109) and protection of mitochondria against intracellular Ca^2+^ overload (110). In addition, several studies have revealed the neuroprotective effects of PROG in experimental crush-induced injury, mediated by increases in brain derived neurotrophic factor (BDNF) (111), preventing secondary neuronal loss (112), as well as by restoring impaired expression of choline acetyltransferase and Na, K-ATPase subunits (113). The neuroprotective effects of PROG may be mediated through its metabolite ALLO: in experimental injury models, ALLO levels increased after PROG administration, and the neuroprotective effect of PROG was diminished by inhibition of ALLO biosynthesis (114, 115).

On the other hand, another plausible mechanism by which neurosteroids could exert neuroprotective effects involves their capacity to modulate polyunsaturated fatty acid (PUFA) metabolism. As a major component of neuron membranes in the brain, long-chain PUFAs play a key role in the maintenance of brain functions, and dysregulation of PUFAs has consistently been observed in SZ (27, 29, 32). Estradiol is able to enhance the synthesis of linolenic acid elongation products, including eicosapentaenoic acid, docosapentaenoic acid, and docosahexaenoic acid in neuroblastoma cells, thereby suggesting that neurosteroids may regulate PUFA synthesis (116). In addition, PROG treatment counteracted several parameters related to decreased synaptic plasticity (BDNF, syntaxin-3, growth associated protein-43, myelin-associated glycoprotein, etc.), and membrane stability (4-hydroxynonenal and secreted phospholipases A_2_) resulting from n-3 PUFA deficiency, which can suggest potential targets for therapeutic applications (12).

### Ameliorating Stress Response

The neuroendocrine response to stress is mediated through the hypothalamic–pituitary–adrenal (HPA) axis (117). Accumulating evidence suggests that the perception of stress by patients with SZ is sufficiently altered so as to lead to a more frequent activation of the primary stress response, namely hyperactivity of the HPA axis (118). It is likely that HPA axis dysfunction not only contributes to the manifestation and exacerbation of symptoms (119) but also to the pattern of poor physical health and premature mortality in SZ patients (117).

The effects of neurosteroids on the stress response may be associated with negative feedback and self-protective mechanisms (21). Acute stress is stimulated in animal models by mild carbon dioxide exposure, foot shock, or forced swimming; these manipulations transiently change the levels of several neurosteroids in both the brain and plasma. Along with corticosterone, an indicator of HPA activation in rodents, levels of PREG, PROG, THDOC, and ALLO are significantly increased in the brain and plasma of acutely stressed animals (120–125). Interestingly, inhibition or complete blockade of GABAergic neurotransmission mimicked the behavioral and physiological effects of stress in rodents, such as inducing anxiety-like behavior (126) and increasing brain and plasma levels of ALLO, THDOC, and cortisol (40, 122, 123). Such effects, however, can be reversed by pretreatment with a positive modulator of the GABA_A_ receptor (122, 123).

It is also possible that a feedback mechanism involving stress-induced increases of ALLO and THDOC could antagonize the suppressive effect of stress on GABAergic transmission (2, 120, 127). Since it is well documented that GABAergic transmission exerts inhibitory effects on HPA axis activity, a diminished GABAergic tone may contribute to the hyperactivity of HPA axis (120). Therefore, increased concentrations of ALLO and THDOC would result in an upregulation of the GABAergic tone, which ameliorates the HPA axis activity.

The effects of chronic stress on neurosteroid concentrations differ from those of acute stress. Mild chronic stress, induced in rodents by social isolation or a combination of several unpredictable stimuli, can cause a significant decrease in PREG, PROG, ALLO, and THDOC in both the brain and plasma (128–131), whereas DHEA levels are unaffected (128). Neurosteroid alterations were non-significant after a 48-h short term of chronic stress, but after a long-term chronic stress, decrements were shown after 7 days and the trend persisted for 30 days (128). Chronic stress was also related to a decrease in GABAergic signaling (128), which could lead to the loss of inhibition of HPA axis activity (132, 133).

## Potential Roles of Neurosteroids in SZ

**Figure 4:**
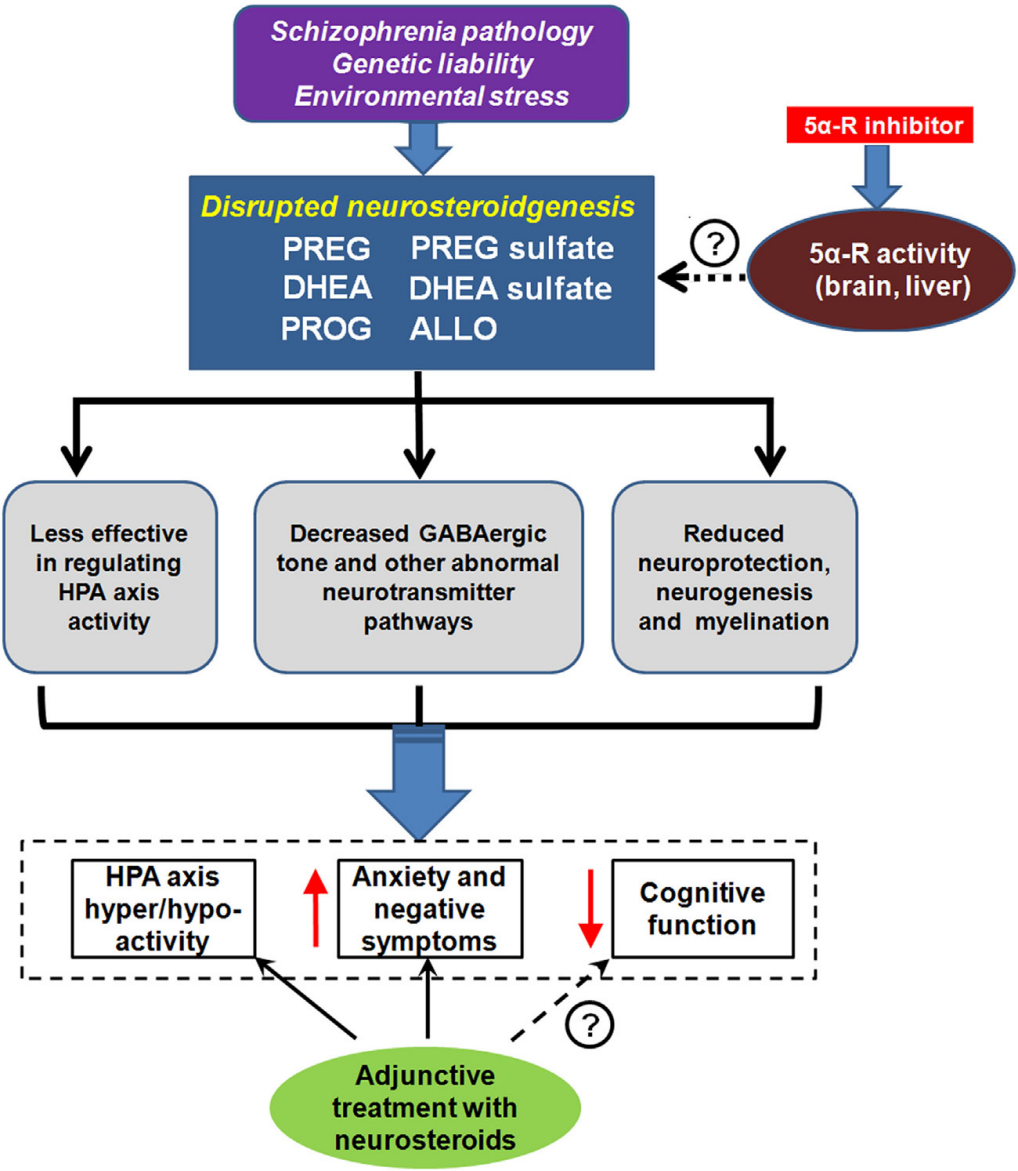
Neurosteroids and schizophrenia (SZ). Unknown SZ pathology, genetic liability, and/or environmental stress may lead to disrupted neurosteroidgenesis, which is manifested by abnormalities in several key neurosteroids, including pregnenolone (PREG), PREG sulfate, dehydroepiandrosterone (DHEA), DHEA sulfate, progesterone (PROG), and allopregnanolone (ALLO). This disruption is associated with dysregulation of the hypothalamic–pituitary–adrenal (HPA) axis, aggravated anxiety, and negative symptoms, as well as impairment in cognition. Some of the symptom domains can be reversed with neurosteroid argumentation. 5α-reductase (5α-R) is a rate-limiting enzyme involved in neurosteroid metabolism. Recent pharmacological studies revealed that the inhibition of 5α-R activity by 5α-R inhibitor could prevent quick exhaustion of the aforementioned neurosteroids. Thus, 5α-R may be a new promising target for SZ treatment. ↑ indicates increased and ↓ indicates decreased.

Accruing evidence from animal models of SZ as well as from patients with SZ suggests that certain key neurosteroids and selected metabolites may play a role in the pathophysiology and therapeutics of SZ (21). The initial findings indicate that PREG administration result in elevations of PS in rodent brain and plasma (134) and of PS and DHEAS in serum of human subjects (135). In addition, the concentrations of downstream metabolites PROG and ALLO in human serum are increased following PREG administration (135). Given the various mechanisms of action that neurosteroids have on the brain, the potential role of neurosteroids of the PREG metabolic pathway should be explored in the context of SZ (Figure 4).

### Neurosteroid Deficits in SZ Patients

Schizophrenia has been linked to a variety of aberrant functions related to HPA axis (117, 136), dopaminergic signaling (137), glutamatergic system (26) and GABAergic system (138). Interestingly, neurosteroids are able to modulate these deficits as well as the sigma-1 system, directly and/or indirectly (99), and thus may be involved in the pathophysiology of this disabling illness. Regulation of these neurosteroids may also contribute to the therapeutic benefits of antipsychotics, especially those which act on the GABA_A_ receptor complex (139, 140).

#### Pregnenolone–Progesterone–Allopregnanolone Pathways

Converging evidence indicates a pattern of reduced PROG and its related endogenous steroids in SZ. Lower levels of serum PREG (141) were found in SZ patients as compared with healthy controls (HC). Moreover, metabolic stress induced by injection of 2-deoxyglucose caused a significantly greater increase in plasma PROG of SZ patients than HC (142). Preliminary evidence suggests that plasma ALLO may be decreased in nonmedicated first-episode SZ patients (135). Evidence from a postmortem study showed a decrement in ALLO levels in the parietal cortex of SZ patients compared to HC (8). However, inconsistent findings were also reported, showing that plasma PROG levels may be lower, or similar in SZ patients when compared to HC (143–145).

Finally, a proof-of-concept trial was conducted with adjunctive PREG administered to SZ patients, with cognition and negative symptoms being the outcomes of interest (135). Increases in levels of serum PREG and ALLO predicted Brief Assessment of Cognition in schizophrenia (BACS) composite scores after 8 weeks of treatment with PREG. In addition, baseline PREG, PS, and ALLO levels were inversely correlated with the improvement in MATRICS Consensus Cognitive Battery (MCCB) composite scores, lending further support to the notion that neurosteroids play a significant role in SZ-related cognitive dysfunction (135).

#### Transformation from DHEA to DHEA Sulfate

Abnormalities in the conversion of DHEA to DHEAS have also been implicated in SZ. The earlier studies reporting decreased plasma DHEA levels in SZ patients (146, 147) were questionable due to methodology issues. However, the later studies with improved methodology have shown elevated levels of plasma DHEA and DHEAS in severely psychotic male subjects (148), chronic SZ patients under treatment (149), and first-episode drug-naïve patients (150). A recent finding suggests that increased serum DHEAS may exist only in the first-episode but not in subsequent recurrences of male SZ patients (151), which is not in accordance with the earlier study using a state-of-art gas chromatography-mass spectrometry method (145).

As compared to DHEA or DHEAS alone, it has been suggested that the ratio of DHEA/cortisol or DHEAS/cortisol may be more useful indicators (152). Ritsner et al. (153) reported that the ratios of DHEA/cortisol and DHEAS/cortisol were higher in SZ patients than in HC, whereas the ratio of DHEA/cortisol remained unchanged in another cohort of SZ patients (154). Moreover, lower levels of circulating DHEAS (150), DHEA and the ratio of DHEA/cortisol were correlated with higher severity of symptomatology in SZ patients (155). Consequently, high levels of circulating DHEA and/or DHEAS may reflect a better functioning in SZ patients, though it is paradoxical that several abovementioned studies showed that this population has higher DHEA and/or DHEAS levels than matched controls (148–150).

In sum, dysregulated neurosteroids may be related to some physiological and/or functional alterations in SZ. First, decreases in brain levels of ALLO, which positively modulates GABAergic transmission, in combination with elevations of DHEA and DHEAS, which negatively modulated GABAergic activity, could lead to impairments of GABAergic function that are observed in SZ. Second, excessive serotonergic activity in SZ patients (156) may result from low levels of PROG and ALLO, antagonists of the 5-HT_3_ receptor (157). Third, neurosteroids targeting sigma-1 receptors may be beneficial in the treatment of SZ patients (158). For instance, increases in DHEA and DHEAS levels [positive sigma-1 receptor modulators (98, 99)], in combination with decreased concentrations of PROG [an antagonist of sigma-1 receptor (99)], may play a role in the pathophysiology of SZ. In addition, it has been shown that DHEA and DHEAS can stimulate NMDA receptor activity, which may be in part responsible for the hyperactivity and subsequently reduced density of NMDA receptor in SZ (159).

## Therapeutic Properties of Neurosteriods

### Neurosteroids in Experimental Models

#### Effects on Learning and Memory in Animal Behavioral Models

It is well known that cognitive functions profoundly impact on the outcome and long-term quality of life in SZ patients (17, 18, 160–162). The neurosteroids appear to exhibit positive effects on learning and memory (17, 18, 163, 164) at physiologically relevant concentrations (165). Further, the improvements in learning and memory dysfunctions have been demonstrated in several behavioral animal models after treatment with neurosteroids (16, 166, 167).

Selective NMDA receptor antagonists are often used to model the putative state of SZ and its therapeutic outcome (168, 169). Since NMDA receptor antagonists such as ketamine not only induce psychotic-like symptoms in healthy volunteers but also exacerbate psychotic symptoms in SZ patients (170, 171), these neurosteroid actions may be of particularly relevance to the pathophysiology of SZ, and to the treatment of cognitive symptoms. Specifically, PS administration can prevent the deficits in learning and memory resulting from NMDA receptor antagonists, including MK-801, dizocilpine and scopolamine (172–175). Like PS, the GABAergic ALLO is also able to ameliorate the MK-801-induced behavioral effects (176, 177). In short, several neurosteroids have played a role in the reversal of MK-801-induced behavioral changes, which may provide us with therapeutic targets and/or monitoring indices for SZ patients.

#### Antipsychotic-Like Properties of Neurosteroids

PREG and its metabolites exhibit antipsychotic and cognitive enhancing properties, implying that the lower levels of these neurosteroids may lead to a higher vulnerability to psychosis, whereas the higher levels may reflect a better therapeutic outcome (166).

In general, atypical antipsychotic drugs appear to be better than typical antipsychotics to treat cognitive deficits in SZ (178). In several behavioral paradigms, PROG and ALLO have demonstrated similar antipsychotic-like properties. Increases of PROG and ALLO levels in the brain were observed after clozapine (6, 41, 167, 179) and olanzapine (7, 179, 180) administration, but not haloperidol (41) administration. Intracerebroventricular administration of ALLO inhibited motor hyperactivity induced by amphetamine (181). Intraperitoneal and intracerebroventricular administration of PROG and ALLO, respectively, could produce inhibition of the conditioned avoidance response in rodents (11). Similar effects were also observed following intraperitoneal injection of antipsychotics olanzapine, risperidone, and haloperidol (11, 182). Such antipsychotic effects are likely owing to the interactions among these neurosteroids, GABAergic system, and dopaminergic system as discussed in Section “Modulation of Dopaminergic Activity and Sigma Receptors.”

### Neurosteroid Trial Studies in Patients

Since PREG and DHEA exhibit antipsychotic properties, efforts have been undertaken to utilize these neurosteroids as potential adjunctive drugs for treatment of SZ as outlined in Table 1.

**Table 1:** The main indices and results from clinical trials using dehydroepiandrosterone (DHEA) and pregnenolone (PREG) as an augmentation therapy in schizophrneia and schizoaffective disorders.

Reference	Patients characteristics	Antipsychotics	Subjects (daily dosage)			Duration (weeks)	Significant effects on different aspects as compared to placebo			
			DHEA	PREG	Placebo		Symptoms	Cognition	Side effects	Quality of life
Strous et al. (183)	SZ inpatients, longer than 2-year duration, prominent negative symptoms	FGAs, SGAs	15 (100 mg)	–	12	6	Negative, depressive and anxiety symptoms	No data	No data	No data
Nachshoni et al. (184)	Inpatients with SZ/SA	FGAs, SGAs	15 (100 mg)	–	15	1	No effect	No data	Extrapyramidal symptoms particularly Parkinson-like symptoms	No data
Strous et al. (185)	Stable chronic SZ patients	Olanzapine	16 (150 mg)	–	15	12	No effect	No effect	No effect	No effect
Ritsner et al., Strous et al., and Ritsner and Strous (186–188)	Inpatients and outpatients with SZ	FGAs, SGAs	55 (200 mg)	–	55	6	No effect	Sustained attention, memory and executive function	No effect	No effect
Ritsner et al. (189)	Patients with chronic SZ/SA	FGAs, SGAs	16 (400 mg)	16 (30 mg), 10 (200 mg)	16	8	Positive symptoms^b^	Attention and working memory performance	Extrapyramidal symptoms^c^	No data
Kreinin et al. (190)	Patients with SZ/SA	SGAs	–	8 (500 mg)^a^	9	8	No effect	No effect	No effect	No effect
Marx et al. and Ritsner et al. (135, 191)	Out- and inpatients with recent-onset SZ/SA, with suboptimal response to antipsychotics	FGAs, SGAs	–	25 (50 mg)	27	8	Negative symptoms especially on blunted affect, avolition and anhedonia domain scores	Visual attention deficits	No effect	No data
Marx et al. (192)	Outpatient with SZ treated with an antipsychotic for the previous 8 weeks or longer	FGAs, SGAs	–	56 (500 mg)^a^	55	8	No effect	No effect	No effect	Functional capacity

*^a^Dosing:100 mg/day for 2 weeks, 300 mg/day for 2 weeks, and 500 mg/day for 4 weeks*.

*^b^Significant for PREG augmentation at 30 mg/day*.

*^c^Significant for PREG and DHEA argmentations*.

*SZ, schizophrenia; SA, schizoaffective disorder; FGAs, first-generation antipsychotics; SGA, second-generation antipsychotics*.

During a 6-week randomized, double-blind, placebo-controlled study (183), SZ patients receiving DHEA augmentation (100 mg/day) demonstrated a significant increase in plasma DHEAS levels and concomitant improvements in negative symptoms, but not in depressive or anxiety symptoms. Using a similar clinical intervention protocol, Nachshoni et al. (184) also observed an improvement of extrapyramidal symptoms (EPS) in SZ patients following add-on treatment of DHEA. However, a subsequent study by the same research group failed to demonstrate an overall improvement in symptomatology of chronic SZ patients following an add-on treatment of DHEA (150 mg/day) for 12-weeks (185). These discrepancies might be due to a number of methodological differences, which could be resolved by a randomized, double-blind, placebo-controlled, crossover trial (186–188). However, a crossover analysis failed to demonstrate improvements on symptom severity, side effects, or quality of life in SZ patients following add-on DHEA treatment (186, 187). Using the multiple regression method for predicting sustained attention, memory, and executive function scores (188), however, these same data sets (186, 187) suggested that peripheral DHEAS and androstenedione levels may be utilized as positive predictors, whereas DHEA level as negative predictor for cognitive functioning.

On the other hand, several proof-of-concept clinical trials with add-on treatment of PREG were also reported in SZ patients. Findings (189) suggested that PREG exhibited higher therapeutic efficacy than DHEA in psychotic symptoms and cognitive performance from patients with chronic SZ and schizoaffective disorder. Interestingly, patients that received a low dosage (30 mg/day) of PREG demonstrated a significant amelioration in positive symptoms and EPS side effects, as well as improvements in attention and working memory performance (190, 191), whereas patients that were treated with a high dose (200 mg/day) of PREG showed no differences on the outcome variables during the study period (189).

When add-on dose of PREG was increased to 500 mg/day, SZ or schizoaffective patients treated with PREG demonstrated significantly greater reduction in the Scale for the Assessment of Negative Symptoms scores than patients receiving placebo (135). Elevation of serum PREG and ALLO levels predicted the BACS (192) composite scores at 8 weeks in the PREG-treated group. In addition, baseline PREG, PS, and ALLO levels were inversely associated with improvements in the MCCB (192) composite scores. Recently, a similar clinical intervention protocol, but with a larger cohort, was conducted at different study sites (190, 191). The results of this study further support a therapeutic role for neurosteroids in alleviating negative symptoms and/or cognitive dysfunctions in SZ.

Thus, clinical interventions that target neurosteroid systems merit further investigation as potential novel treatments for SZ. Moreover, new classes of synthetic neurosteroid analogs that have superior bioavailability and safety profiles compared to natural neurosteroids may be advantageous for clinical use (48).

## Future Investigations

To date, convincing evidence has suggested that neurosteroids can play various roles in the etiology and treatment of many stress-related psychiatric disorders, such as anxiety, depression, and SZ (193, 194). In previous studies, only a few neurosteroids of interest were investigated in SZ. In the future, however, it is critical to evaluate more neurosteroids simultaneously in order to test whether they are integrated with each other within the same PREG-PROG-ALLO pathway or across other related pathways. Although the possible interactions between neurosteroids and the GABA_A_ receptor were the primary focus of past studies, interactions of these endogenous molecules with other receptor complexes (e.g., NMDA, dopamine, and sigma-1) also need to be explored in mood disorders and SZ.

Neurosteroids may possess intrinsic antipsychotic properties. Using random controlled trials on different key neurosteroids, several recent pilot studies have already shown promising leads to therapeutic development. However, future studies in larger cohorts addressing the effects of neurosteroids on multiple neurotransmitter receptors and the behavioral consequences of long-term follow-ups are still warranted.

In addition, neuroprotective strategies using exogenous neurosteroids or other compounds (such as 5α-R inhibitor) modulating the biosynthesis of neurosteroids (195, 196) may alleviate some limitations of current antipsychotic drugs, by reducing the cognitive deficits and negative symptoms, as well as by improving functioning and quality of life in patients with SZ.

## Author Contributions

All the authors participated in the writing of the manuscript. HC and JY were the primary writers. TC and XZ conducted a wide literature review of preclinical and clinical studies and contributed to organizing related references. JY was the senior author of the manuscript and provided overall guidance and feedback on revisions of the manuscript.

## Conflict of Interest Statement

The authors declare that the research was conducted in the absence of any commercial or financial relationships that could be construed as a potential conflict of interest.
